# Outcomes of Optical Coherence Tomography-Guided and Angiography-Guided Primary Percutaneous Coronary Intervention in Patients with ST-Segment Elevation Myocardial Infarction

**DOI:** 10.31083/j.rcm2512444

**Published:** 2024-12-18

**Authors:** Jiannan Li, Xiaoli Wang, Runzhen Chen, Peng Zhou, Chen Liu, Li Song, Yi Chen, Hongbing Yan, Hanjun Zhao

**Affiliations:** ^1^Department of Cardiology, Fuwai Hospital, National Center for Cardiovascular Diseases, Peking Union Medical College & Chinese Academy of Medical Sciences, 100037 Beijing, China; ^2^Department of Cardiology, Dongguan Cardiovascular Research Institute, Dongguan Songshan Lake Central Hospital, Guangdong Medical University, 523770 Dongguan, Guangdong, China; ^3^Fuwai Hospital, Chinese Academy of Medical Sciences, 510000 Shenzhen, Guangdong, China

**Keywords:** optical coherence tomography, ST-segment elevation myocardial infarction, outcomes, angiography

## Abstract

**Background:**

Despite the administration of timely reperfusion treatment, patients with acute myocardial infarction have a high mortality rate and poor prognosis. The potential impact of intraluminal imaging guidance, such as optical coherence tomography (OCT), on improving patient outcomes has yet to be conclusively studied. Therefore, we conducted a retrospective cohort study to compare OCT-guided primary percutaneous coronary intervention (PCI) versus angiography-guided for patients with ST-segment elevation myocardial infarction (STEMI).

**Methods:**

This study enrolled 1396 patients with STEMI who underwent PCI, including 553 patients who underwent OCT-guided PCI and 843 patients who underwent angiography-guided PCI. The clinical outcome was a composite of cardiovascular death, myocardial infarction, admission due to heart failure, stroke, and unplanned revascularization at the 4-year follow-up.

**Results:**

The prevalence of major adverse cardiovascular events in OCT-guided group was not significantly lower compared to those without OCT guidance after adjustment (unadjusted hazard ratio (HR), 1.582; 95% confidence interval (CI), 1.300–1.924; *p* < 0.001; adjusted HR, 1.095; adjusted 95% CI, 0.883–1.358; *p* = 0.409). The prevalence of cardiovascular death was significantly lower in patients with OCT guidance compared to those without before and after adjustment (unadjusted HR, 3.303; 95% CI, 2.142–5.093; *p* < 0.001; adjusted HR, 2.025; adjusted 95% CI, 1.225–3.136; *p* = 0.004).

**Conclusions:**

OCT-guided primary PCI used to treat STEMI was associated with reduced long-term cardiovascular death.

## 1. Introduction

Optical coherence tomography (OCT) is an intravascular imaging modality based on infrared light imaging [1], which has greater resolution than intravascular ultrasound, providing more advantages for evaluating plaque morphology and the immediate effect of stent deployment [2]. Compared with coronary angiography-guided percutaneous coronary intervention, OCT-guided percutaneous coronary intervention (PCI) results in a larger minimum stent area and reduces the incidence of coronary dissection and stent malposition [3]. OCT provides clearer images of plaque characteristics than other intravascular modalities, such as intravascular ultrasound (IVUS). However, both OCT- and IVUS-guided PCI can improve patient prognoses compared with coronary angiography [4, 5]. OCT-guided PCI offers no significant improvement over IVUS-guided PCI in decreasing the incidence of major adverse cardiovascular events (MACEs) at 1 year [6], including in guiding interventions for complex coronary lesions [7]. However, the incidence of major procedural complications is significantly lower among patients undergoing OCT-guided treatment than those undergoing IVUS-guided treatment [6].

Nevertheless, the impact of OCT guidance on the long-term prognosis of patients remains controversial among different studies. Previous clinical investigations have demonstrated that using OCT in PCI significantly reduces MACEs, cardiovascular death, and revascularization [8], especially in complex coronary artery disease [4, 9]. However, using OCT-guided PCI for non-complex lesions did not significantly differ from using angiography guidance in clinical outcomes at 12 months [3]. However, more dedicated studies are needed to confirm the superiority of OCT over coronary angiography. Therefore, this study explored whether OCT examination can improve the prognosis of ST-segment elevation myocardial infarction (STEMI) patients. In this study, long-term clinical outcomes were compared between patients who received PCI treatment under OCT guidance and those who received PCI treatment under angiography guidance in a retrospective cohort.

## 2. Methods

### 2.1 Population

**Fig. 1.**
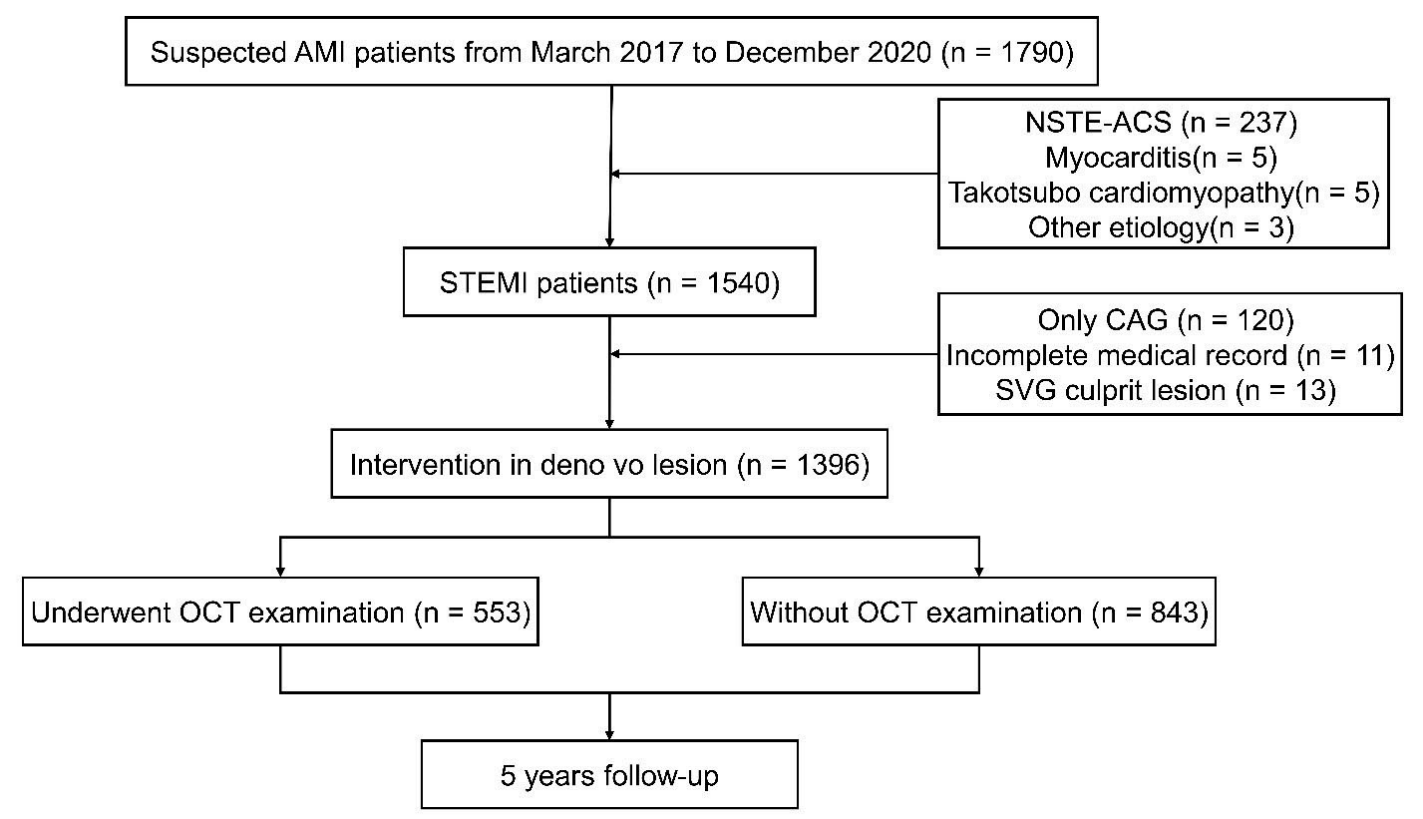
**Study flowchart**. AMI, acute myocardial infarction; STEMI, ST segment elevation myocardial infarction; NSTE-ACS, non ST segment elevation acute coronary syndrome; CAG, coronary angiography; SVG, saphenous vein graft; OCT, optical coherence tomography.

This retrospective study consecutively enrolled 1790 suspected acute myocardial infarction (AMI) patients from March 2017 to December 2020 at Fuwai Hospital (Beijing). Patients aged 18 years or older who underwent emergent coronary angiography due to an AMI diagnosis (symptom onset ≤24 h before presentation) were included. After excluding patients with non-ST-segment elevation myocardial infarction (NSTEMI) (n = 237), myocarditis (n = 5), takotsubo cardiomyopathy (n = 5), other etiologies (n = 3), and STEMI patients who only had coronary angiography (CAG) examinations (n = 120), incomplete medical records (n = 11) or saphenous vein graft (SVG) culprit lesions (n = 13), 1396 patients were included in the final analysis (Fig. 1). Among these patients, 553 underwent OCT examinations, while 843 did not. Demographic data, risk factors for coronary heart disease, and laboratory results were collected and recorded based on the medical history and initial laboratory examination of each patient after admission. A professional PCI operator provided the coronary angiography and interventional data. The baseline characteristic definitions are provided in the **Supplementary Methods**. STEMI was defined as continuous chest pain lasting more than 30 minutes, ST-segment elevation greater than 0.1 mV in at least two contiguous leads or a new left bundle-branch block on the 18-lead electrocardiogram (ECG), and an elevated troponin I level [10]. Follow-up information on MACEs, including cardiovascular death, myocardial infarction, heart failure, stroke, and unplanned revascularization, was collected through telephone interviews and outpatient visits by trained cardiologists at scheduled intervals, including one, six, and twelve months after discharge, followed by annual assessments. This study was approved by the Ethics Committee of Fuwai Hospital (No. 2017-866) in accordance with the Declaration of Helsinki. All patients provided signed informed consent.

### 2.2 Acquisition of OCT Images

Patients were administered 300 mg of aspirin, 180 mg of ticagrelor, 600 mg of clopidogrel, and 100 IU/kg heparin before the interventional procedure. The percutaneous coronary intervention was performed via radial or femoral access. The decision to conduct an OCT examination before or after stenting depended on the judgment of the operator and the condition of the patient. Thrombus aspiration reduced the thrombus burden and restored antegrade coronary flow. After antegrade blood flow was restored, OCT images of the culprit lesions were acquired using the frequency domain ILUMIEN OPTIS OCT system and a dragonfly catheter (St. Jude Medical, Westford, MA, USA), according to a previously described intracoronary imaging technique [11]. Three independent investigators analyzed all anonymized OCT images and other data using a St Jude OCT Offline Review Workstation. The definitions of pre- and post-interventional OCT characteristics are provided in the **Supplementary Material**.

### 2.3 Statistical Analysis

Continuous data are presented as the mean ± SDs or medians (interquartile ranges). Student’s *t*-test or nonparametric tests were used for statistical comparisons. Categorical variables are presented as counts (percentages); comparisons between groups were performed using the χ^2^ or Fisher’s exact tests. Kaplan–Meier curves, log-rank tests, and Cox proportional hazards regressions were conducted to compare MACE risks between groups. The multivariable Cox regression was adjusted for traditional cardiovascular risk factors, including age, sex, diabetes mellitus, hypertension, stroke, previous myocardial infarction, chronic kidney disease, Killip level, creatine, cardiac troponin I (c-TnI), N-terminal pro-B-type natriuretic peptide (NT-proBNP), lesion diameter and length, thrombus aspiration, left ventricle ejection fraction, and anterior wall infarction. Subgroup analysis for the primary outcome was performed based on age (≥60 *vs*. <60 years), sex, diabetes, hypertension, smoking, anterior wall infarction, Killip level (I *vs*. II/III/IV), American Heart Association (AHA) lesion type (A/B *vs*. C), ejection fraction (EF, ≥50% *vs*. <50%), triglycerides (TGs, ≥1.7 *vs*. <1.7 mmol/L), low-density lipoprotein cholesterol (LDL-C, ≥2.6 *vs*. <2.6 mmol/L), and high-sensitivity C-reactive protein (hs-CRP, >3 *vs*. ≤3 mg/dL) using Cox regression with multiple adjustments for all baseline variables. A two-tailed *p* < 0.05 was considered statistically significant. Statistical analyses were conducted using SPSS software (version 26.0; IBM Corp., Armonk, NY, USA) and R statistical packages (http://www.r-project.org/).

## 3. Results

The baseline characteristics of the two groups are shown in Table 1. Patients in the group without OCT guidance were more likely to be older, and this group had a greater proportion of patients with previous myocardial infarction, ischemic stroke, and chronic kidney disease. These patients also had higher creatinine, NT-proBNP, and Killip II–IV levels and lower left ventricular ejection fraction (LVEF) levels. The OCT-guided group had a greater proportion of anterior wall myocardial infarction, larger implanted stents, and higher application rates of aspirin, statin, angiotensin-converting enzyme inhibitor/angiotensin receptor blocker (ACEI/ARB) use than the group without OCT guidance.

**Table 1.** **Baseline characteristics**.

Variables			Total (n = 1396)	OCT guidance		*p*-value
				With (n = 553)	Without (n = 843)	
Demographic data						
	Age (years)		60.2 ± 12.4	58.2 ± 11.8	61.6 ± 12.7	<0.001
	Males		1134 (81.2%)	464 (83.9%)	670 (79.5%)	0.038
	BMI (kg/m^2^)		25.9 ± 3.7	26.0 ± 3.4	25.8 ± 3.8	0.316
Risk factors						
	Diabetes mellitus		459 (32.9%)	163 (29.5%)	296 (35.1%)	0.028
	Hypertension		888 (63.6%)	333 (60.2%)	555 (65.8%)	0.033
	Hyperlipidemia		1249 (89.5%)	499 (90.2%)	750 (89.0%)	0.451
	Ischemic stroke		188 (13.5%)	57 (10.3%)	131 (15.6%)	0.005
	Previous MI		217 (15.5%)	55 (9.9%)	162 (19.2%)	<0.001
	Chronic kidney disease		98 (7.0%)	19 (3.4%)	79 (9.4%)	<0.001
	Smoker		1003 (72.2%)	410 (74.4%)	593 (70.7%)	0.129
Clinical indicator						
	Killip II–IV level		191 (13.7%)	44 (8.0%)	147 (17.4%)	<0.001
	LVEF (%)		53.3 ± 7.6	54.5 ± 6.1	52.5 ± 8.3	<0.001
	hs-CRP (mg/L)		6.2 (2.2–11.0)	5.9 (2.4–10.8)	6.6 (2.1–11.1)	0.260
	Creatinine (mmol/L)		89.6 ± 38.5	84.6 ± 38.0	92.8 ± 38.5	<0.001
	HbA1c (%)		6.7 ± 1.6	6.6 ± 1.6	6.7 ± 1.6	0.278
	Triglyceride (mmol/L)		1.4 (1.0–2.1)	1.4 (1.0–2.0)	1.5 (1.1–2.1)	0.099
	LDL-C (mmol/L)		2.7 ± 0.9	2.7 ± 0.9	2.7 ± 0.9	0.889
	HDL-C (mmol/L)		1.0 (0.9–1.2)	1.1 (0.9–1.2)	1.0 (0.9–1.2)	0.165
	c-TnI (ng/mL)		1.0 (0.1–5.3)	0.9 (0.1–4.7)	1.1 (0.1–6.0)	0.013
	NT-proBNP (pg/mL)		262.4 (65.8–922.6)	191.7 (50.4–579.1)	311.7 (87.4–1265.3)	<0.001
	Anterior wall infarction		639 (45.8%)	275 (79.4%)	364 (43.2%)	0.016
Angiographic data						
	Culprit lesion					0.003
		LM	9 (0.6%)	0 (0)	9 (1.1%)	
		LAD	639 (45.8%)	275 (49.7%)	364 (43.2%)	
		LCX	181 (13.0%)	57 (10.3%)	124 (14.7%)	
		RCA	567 (40.9%)	221 (40.0%)	346 (41.0%)	
	AHA lesion type					0.063
		A	30 (2.1%)	8 (1.4%)	22 (2.6%)	
		B1	144 (10.3%)	51 (9.2%)	93 (11.0%)	
		B2	277 (19.8%)	98 (17.7%)	179 (21.2%)	
		C	945 (67.7%)	396 (71.6%)	549 (65.1%)	
	Lesion length		23.0 (16.0–32.0)	25.0 (18.0–34.0)	22.0 (15.0–30.0)	<0.001
	Lesion diameters		3.0 (2.5–3.5)	3.0 (2.8–3.5)	2.8 (2.5–3.3)	<0.001
	Stenosis (%)		96.8 ± 7.2	96.7 ± 8.4	96.9 ± 6.3	0.642
	Pre-intervention TIMI 0		888 (63.6%)	356 (64.4%)	532 (63.1%)	0.630
	Multivessel lesion		1039 (74.4%)	400 (72.3%)	639 (75.8%)	0.146
	Stent diameters		3.1 ± 0.5	3.2 ± 0.5	3.1 ± 0.5	<0.001
	Stent length		30.4 ± 15.4	31.7 ± 14.8	29.4 ± 15.8	0.010
	Thrombus aspiration		538 (38.5%)	297 (53.7%)	241 (26.8%)	<0.001
	Pre-dilatation		1274 (91.3%)	480 (86.8%)	794 (94.2%)	<0.001
	Post-dilatation		1160 (83.1%)	504 (91.1%)	656 (77.8%)	<0.001
	Post-intervention TIMI					<0.001
		0	20 (1.4%)	0 (0)	20 (2.4%)	
		1	8 (0.6%)	0 (0)	8 (0.9%)	
		2	26 (1.9%)	4 (0.7%)	22 (2.6%)	
		3	1342 (96.1%)	549 (99.3%)	793 (94.1%)	
Discharge medication						
	Aspirin		1330 (95.3%)	537 (97.1%)	793 (94.1%)	0.009
	Ticagrelor		693 (49.6%)	293 (53.0%)	400 (47.4%)	0.043
	Clopidogrel		683 (48.9%)	258 (46.7%)	425 (50.4%)	0.169
	ACEI/ARB		1003 (71.8%)	419 (75.8%)	584 (69.3%)	0.008
	β-blocker		1205 (86.3%)	492 (89.0%)	713 (84.6%)	0.020
	Statin		1320 (94.6%)	536 (96.9%)	784 (93.0%)	0.002

Continuous data are presented as the mean ± SD or median (interquartile range), and categorical variables are presented as a %. BMI, body mass index; MI, myocardial infarction; HDL-C, high-density lipoprotein cholesterol; LDL-C, low-density lipoprotein cholesterol; hs-CRP, high-sensitivity C-reactive protein; c-TnI, cardiac troponin I; ACEI, angiotensin-converting enzyme inhibitor; LVEF, left ventricular ejection fraction; ARB, angiotensin receptor blocker; OCT, optical coherence tomography; HbA1c, hemoglobin A1c; NT-proBNP, N-terminal pro-B-type natriuretic peptide; LM, left main; LAD, left Anterior descending artery; LCX, left circumflex artery; RCA, right coronary artery; AHA, American Heart Association; TIMI, thrombolysis in myocardial infarction.

The OCT characteristics are shown in **Supplementary Table 1**. A total of 538 (97.3%) patients underwent pre-interventional OCT examinations, while 275 (49.7%) underwent post-interventional OCT examinations. Among the patients with pre-intervention OCT images, 191 (35.5%) were diagnosed with plaque rupture, 173 (32.2%) with plaque erosion, and 18 (3.3%) with calcified nodules. Twelve (2.2%) patients were categorized as having coronary spasms, embolism, or severe stenosis. Forty-one (7.6%) patients were diagnosed with stent thrombosis. However, the plaque phenotypes of 103 (19.1%) patients remained undetermined because massive thrombi overlapped with the underlying plaque. Among the 275 patients who underwent post-intervention OCT, 151 (54.9%) exhibited plaque prolapse, and 113 (41.1%) had stent malapposition; these patients underwent post-dilatation. Additionally, stent edge dissection was found in 38 patients (13.8%).

**Fig. 2.**
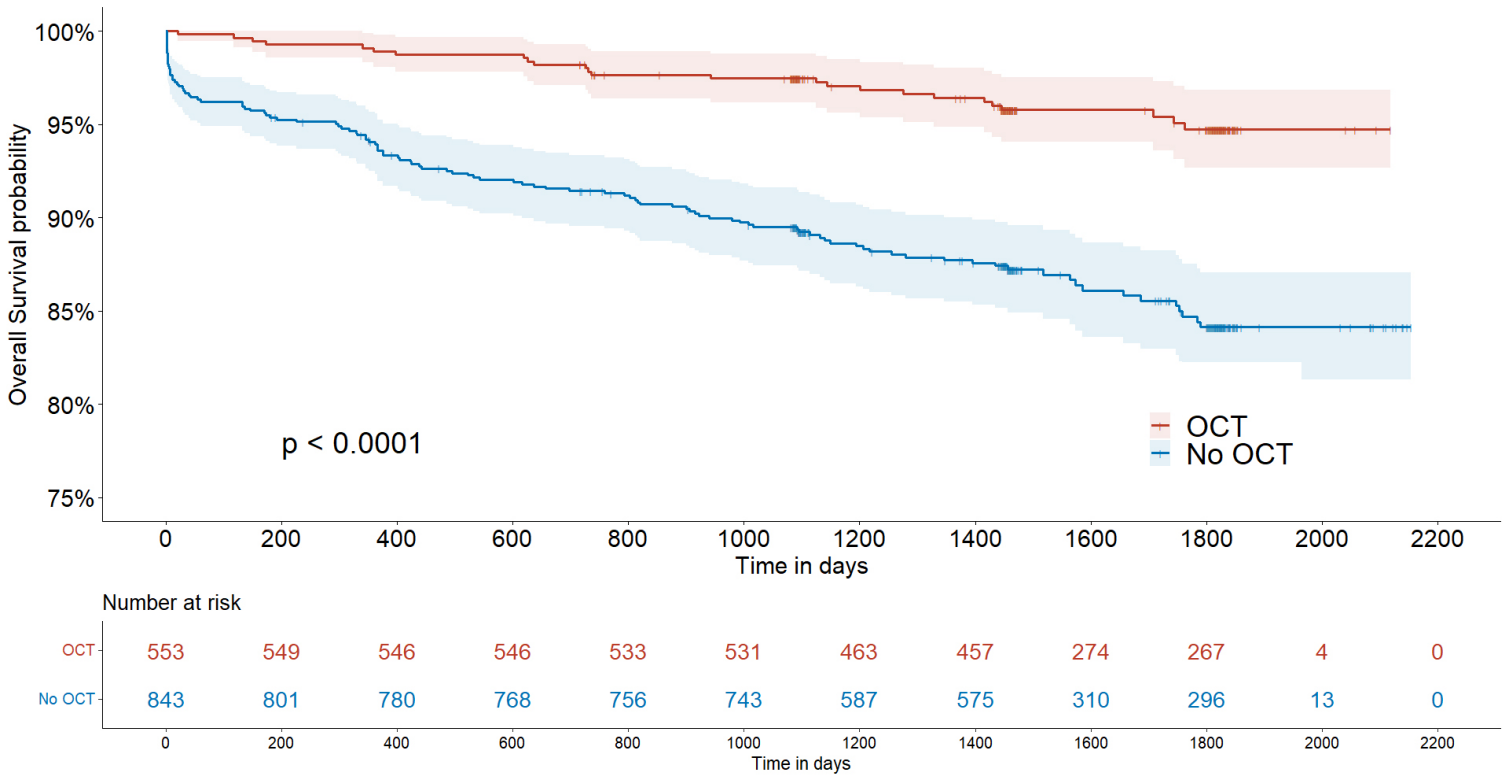
**Kaplan–Meier survival curves in patients with and without OCT guidance**. OCT, optical coherence tomography.

During follow-up, the prevalence of MACEs, including cardiovascular death, myocardial infarction, admission due to heart failure, stroke, and unplanned revascularization, was significantly lower in patients with OCT guidance compared to those without OCT guidance (hazard ratio (HR): 1.582; 95% confidence interval (CI): 1.300–1.924; *p* < 0.001) (Table 2). However, this significant difference was not preserved after adjusting for traditional risk factors and significantly different variables between the two groups in the univariate analysis (HR: 1.095; 95% CI: 0.883–1.358; *p* = 0.409) (Table 2). The occurrence of cardiovascular death was significantly lower in patients with OCT guidance compared to those without, according to both univariate and multivariate analyses (unadjusted HR, 3.303; 95% CI, 2.142–5.093; *p* < 0.001; adjusted HR, 2.025; adjusted 95% CI, 1.225–3.136; *p* = 0.004) (Table 2). The 5-year Kaplan–Meier curves for event-free survival rates are shown in Fig. 2. Additionally, subgroup analysis revealed that patients who underwent OCT examination had significantly lower mortality in each subgroup (**Supplementary Table 2**).

**Table 2.** **Cox regression analyses of MACEs in patients with or without OCT guidance**.

Endpoint		Unadjusted (95% CI)*	*p*-value	Model 1 (95% CI)*	*p*-value	Model 2 (95% CI)*	*p*-value	Model 3 (95% CI)*	*p*-value
MACEs composite		1.582 (1.300–1.924)	<0.001	1.514 (1.243–1.844)	<0.001	1.412 (1.156–1.724)	<0.001	1.095 (0.883–1.358)	0.409
	Death	3.303 (2.142–5.093)	<0.001	2.588 (1.670–4.010)	<0.001	2.372 (1.525–3.691)	<0.001	2.025 (1.225–3.136)	0.004
	MI	1.254 (0.761–2.067)	0.374	1.202 (0.727–1.989)	0.473	1.049 (0.630–1.747)	0.853	0.989 (0.575–1.702)	0.969
	HF	1.994 (1.030–3.863)	0.041	1.914 (0.985–3.720)	0.055	1.735 (0.883–3.411)	0.110	1.305 (0.630–2.704)	0.473
	Stroke	1.326 (0.821–2.142)	0.248	1.285 (0.794–2.082)	0.308	1.145 (0.702–1.865)	0.588	0.985 (0.587–1.650)	0.953
	Revascularization	1.224 (0.943–1.589)	0.129	1.227 (0.943–1.595)	0.127	1.139 (0.873–1.487)	0.337	1.079 (0.814–1.430)	0.596

Model 1: adjusted for age and sex; Model 2: adjusted for all factors in model 1 plus diabetes mellitus, hypertension, stroke, previous myocardial infarction, chronic kidney disease; Model 3: adjusted for all factors in model 2 plus Killip level, creatine, c-TnI, NT-proBNP, lesion diameter and length, AHA lesion types, thrombus aspiration, left ventricle ejection fraction, and anterior wall infarction. * Hazard ratio (with OCT guidance *vs*. without OCT guidance). MACE, major adverse cardiovascular event; OCT, optical coherence tomography; MI, myocardial infarction; HF, heart failure; c-TnI, cardiac troponin I; NT-proBNP, N-terminal pro-B-type natriuretic peptide; AHA, American Heart Association.

## 4. Discussion

In this retrospective cohort study, we first demonstrated that patients with STEMI who underwent OCT-guided primary PCI experienced better clinical outcomes, particularly a lower incidence of cardiovascular death, than those who did not receive OCT guidance. These findings provide evidence supporting the superiority of intravascular imaging in treating critical coronary patients.

Compared with intravascular ultrasound, OCT is a more recent imaging modality characterized by superior resolution and greater accuracy in differentiating and characterizing plaque phenotypes, thereby providing more precise stratification and treatment [12]. Moreover, intravascular imaging guidance of coronary stent implantation with either OCT or intravascular ultrasound enhances the safety and effectiveness of PCI [8, 13]. Furthermore, OCT imaging has been shown to be safe, with a previous study revealing that OCT did not increase periprocedural complications, type 4a myocardial infarction, or acute kidney injury [14]. However, the impact of OCT examination on the long-term clinical outcomes of patients through the guidance of interventional treatment remains limited [6]. A prospective cohort study including 214 patients who underwent OCT-guided primary PCI revealed no significant reduction in clinical events with or without OCT guidance at 1 year [15]. A recent prospective, randomized, single-anonymized trial with 1233 patients assigned to undergo OCT-guided PCI demonstrated no apparent difference in the percentage of patients with target-vessel failure at 2 years [4]. However, in patients with complex coronary artery lesions, intravascular OCT-guided PCI was associated with a lower risk of death from cardiac disorders, target vessel-related myocardial infarction, or clinically driven target vessel revascularization compared to angiography-guided PCI [16]. Moreover, comparisons of clinical outcomes demonstrated that OCT-guided PCI was non-inferior to IVUS [13, 17, 18]. In another comparative study, OCT-guided PCI of non-complex lesions did not significantly differ from IVUS or angiography guidance regarding clinical outcomes at 12 months [3]. A recent meta-analysis indicated that the estimated absolute effects of intravascular imaging-guided PCI were closely related to baseline risk, which is determined mainly by the severity and complexity of coronary artery disease [9]; however, studies on the long-term clinical outcomes of patients with STEMI who undergo OCT-guided primary PCI are lacking.

In the present study, patients at high risk were not selected for OCT examination, which was performed by an interventional operator who considered factors such as hypotension, malignant arrhythmia, or poor vessel condition. Indeed, a higher Killip level, lower LVEF, and poorer renal function were significantly more common in patients without OCT detection. Moreover, patients in the OCT-guided group had significantly larger culprit lesions, including both length and diameter, than those without OCT guidance. However, the degree of stenosis, rate of thrombolysis in myocardial infarction (TIMI) 0, and number of multivessel lesions were not significantly different between the two groups. However, after adjusting for these risk factors, the mortality rate for patients in the OCT-guided group remained lower than those without OCT guidance, suggesting that OCT guidance might benefit some patients. Interestingly, the length and diameter of stent implantation in the group with OCT examination were greater than those without OCT examination. This suggests that OCT guidance allowed for a more thorough evaluation of the lesional area, significantly improving stent expansion and coronary lesion coverage. OCT-optimized stent deployment significantly reduced the short-term in-segment area of stenosis [19]. The iSIGHT randomized trial revealed that stent expansion with OCT guidance was superior to an optimized angiographic strategy [17]. The principal mechanisms underlying the beneficial effects of OCT guidance have been explored, including a greater minimal stent area, freedom from major edge dissections, and untreated focal reference segment disease [18]. Nevertheless, this research did not investigate the mechanism underlying the superiority of OCT-guided primary PCI in patients with STEMI, which requires further investigation.

A previous study demonstrated that pre-interventional OCT examination allows for developing a strategy by differentiating the plaque phenotype in patients with STEMI [12]. Moreover, pre- and post-interventional OCT guidance of PCI contributed to more precise treatment of culprit lesions [4]. The mechanisms in our study that supported improved prognoses in OCT-guided patients included plaque characterization, more accurate stent implantation, and remediation of immediate post-stenting complications. Intervention operators can obtain more useful information from OCT to guide stenting and postoperative antithrombotic therapy. However, our retrospective study could not differentiate the individual contributions of these factors, which need to be verified by randomized controlled trial in the future.

Our study has several limitations. First, this was a retrospective, single-center cohort study with a moderate sample size. Second, some patients in the angiography group who could not undergo OCT examination due to high risk may have introduced selection bias. Third, some patients with multivessel lesions in our study underwent staged PCI with or without OCT guidance after discharge; however, this information was missing, meaning bias cannot be excluded. Fourth, most OCT images in this study were obtained from patients with large vessels—with diameters larger than 3 mm. Therefore, the effectiveness of OCT in relatively small vessels remains limited. Fifth, because the LVEF of patients was relatively high, the rate of recurrent myocardial infarction was low, which may be biased. Sixth, all patients underwent OCT examination only in their culprit lesion; thus, the plaque characteristics in their non-culprit lesion were unclear. Finally, the mechanism underlying the superiority of OCT-guided primary PCI was not investigated in detail. Hence, further large-scale randomized controlled trials are needed to evaluate the impact of OCT guidance on clinical endpoints.

## 5. Conclusions

Our retrospective study provided evidence that OCT-guided primary PCI in patients with STEMI was associated with a significant reduction in long-term mortality compared with patients without OCT guidance; however, further confirmation of these data is required from prospective randomized studies.

## Data Availability

The datasets used and/or analyzed during this study are available from the corresponding author on reasonable request.

## Supplementary Material

Supplementary material associated with this article can be found, in the online version, at https://doi.org/10.31083/j.rcm2512444.
