# Supplementary material for: Outcomes of Optical Coherence Tomography-Guided and Angiography-Guided Primary Percutaneous Coronary Intervention in Patients with ST-Segment Elevation Myocardial Infarction
Source: Rev Cardiovasc Med. 2024 Dec 18;25(12):444. doi: 10.31083/j.rcm2512444 (PMC11683697; doi:10.31083/j.rcm2512444)
Supplement: Supplementary file 1 [file 2153-8174-25-12-444-s1.docx]

**Methods**

**Definition** Hypertension was defined as blood pressure (BP) ≥ 140/90 mmHg at rest over three measurements or a previous diagnosis of hypertension and current use of antihypertensive drugs^[1]^. Patients were diagnosed with DM if they met one of the following criteria: (i) fasting plasma glucose level ≥ 7.0 mmol/L, (ii) 2-h plasma glucose value ≥ 11.1 mmol/L in the 75-g oral glucose tolerance test (OGTT), and (iii) casual plasma glucose level ≥ 11.1 mmol/L^[2]^. Dyslipidaemia was defined by any of the following parameters^[3]^: total cholesterol level ≥ 5.0 mmol/L, low-density lipoprotein cholesterol (LDL-C) level ≥ 3.0 mmol/L, triglyceride level ≥ 1.7 mmol/L, high-density lipoprotein cholesterol (HDL-C) level < 1.2 mmol/L (in women) or < 1.0 mmol/L (in men). Patients who did not meet the standards for never smokers (never smoked in their lifetime) or former light smokers (stopped smoking at least 15 years ago, with ≤ 10 total pack-years of smoking) were considered current smokers^[4]^. Chronic kidney disease (CKD) was defined as abnormal kidney structure or function for more than 3 months, and end-stage renal disease (ESRD) was the final common pathway for CKD^[5]^. Body mass index was calculated by dividing weight (kg) by the square of height (m^2^). According to previously established criteria^[6]^, plaque rupture was identified by a disrupted fibrous cap with clear cavity formation, and plaque erosion was identified by the presence of an attached thrombus overlying an intact and visualized plaque. Calcified nodules (CNs) were defined as the expulsion of small calcific nodules into the lumen. Plaque prolapse was defined as Protrusion of tissue between struts towards the lumen when the distance from the arc connecting adjacent stent struts to the greatest extent of protrusion was greater than 50μm^[7]^. Stent malappostion was defined as a stent that had a strut with a maximal wall-to-strut distance of ≥200 μm^[8]^. Stent edge dissection was defined as a disruption of the vessel luminal surface with flap at an adjacent site to the stent edge (< 5mm)^[9]^.

**Supplementary Table 1.** OCT characteristics of patients with STEMI

| OCT characteristics | Patients (n=553) |
| --- | --- |
| Pre intervention OCT image | 538 (97.3) |
| Plaque rupture | 191 (35.5) |
| Plaque erosion | 173 (32.2) |
| Calcified nodules | 18 (3.3) |
| Spasm, embolism and severe stenosis | 12 (2.2) |
| In stent thrombosis | 41 (7.6) |
| Massive remaining thrombus | 103 (19.1) |
| Post intervention OCT image | 275 (49.7) |
| Plaque prolapse | 151 (54.9) |
| Stent malapposition | 113 (41.1) |
| Stent edge dissection | 38 (13.8) |

**Supplementary Table 2.** Prognostic impacts of OCT examination on the death across subgroups of established risk factors

| Variables | n (%) | OCT | No OCT | HR (95%CI) | p | p for interaction |
| --- | --- | --- | --- | --- | --- | --- |
| Sex |  |  |  |  |  | 0.854 |
| Male | 1134 (81.23) | 19/464 | 84/670 | 3.34 (2.03 ~ 5.49) | <0.001 |  |
| Female | 262 (18.77) | 6/89 | 30/173 | 3.01 (1.25 ~ 7.26) | 0.014 |  |
| Age |  |  |  |  |  | 0.079 |
| <60 | 654 (46.85) | 2/293 | 22/361 | 9.32 (2.19 ~ 39.63) | 0.003 |  |
| ≥60 | 742 (53.15) | 23/260 | 92/482 | 2.47 (1.56 ~ 3.90) | <0.001 |  |
| LVEF |  |  |  |  |  | 0.012 |
| <50% | 353 (25.29) | 3/107 | 59/246 | 9.89 (3.10 ~ 31.55) | <0.001 |  |
| ≥50% | 1043 (74.71) | 22/446 | 55/597 | 2.02 (1.23 ~ 3.32) | 0.005 |  |
| Smoking |  |  |  |  |  | 0.746 |
| No | 387 (27.84) | 8/141 | 37/246 | 2.96 (1.38 ~ 6.36) | 0.005 |  |
| Yes | 1003 (72.16) | 17/410 | 77/593 | 3.45 (2.04 ~ 5.83) | <0.001 |  |
| Hypertension |  |  |  |  |  | 0.446 |
| No | 508 (36.39) | 9/220 | 28/288 | 2.60 (1.23 ~ 5.52) | 0.013 |  |
| Yes | 888 (63.61) | 16/333 | 86/555 | 3.58 (2.10 ~ 6.11) | <0.001 |  |
| Diabetes |  |  |  |  |  | 0.713 |
| No | 937 (67.12) | 16/390 | 71/547 | 3.48 (2.02 ~ 5.99) | <0.001 |  |
| Yes | 459 (32.88) | 9/163 | 43/296 | 2.91 (1.42 ~ 5.98) | 0.004 |  |
| Killip level |  |  |  |  |  | 0.040 |
| I | 1205 (86.32) | 21/509 | 54/696 | 2.00 (1.21 ~ 3.32) | 0.007 |  |
| II/III/IV | 191 (13.68) | 4/44 | 60/147 | 6.09 (2.21 ~ 16.78) | <0.001 |  |
| hsCRP |  |  |  |  |  | 0.612 |
| ≤3mg/dl | 432 (30.95) | 4/171 | 24/261 | 4.29 (1.49 ~ 12.37) | 0.007 |  |
| >3mg/dl | 964 (69.05) | 21/382 | 90/582 | 3.12 (1.94 ~ 5.02) | <0.001 |  |
| TG |  |  |  |  |  | 0.590 |
| <1.7mmol/L | 880 (63.04) | 15/352 | 73/528 | 3.61 (2.07 ~ 6.29) | <0.001 |  |
| ≥1.7mmol/L | 516 (36.96) | 10/201 | 41/315 | 2.84 (1.42 ~ 5.67) | 0.003 |  |
| LDL-C |  |  |  |  |  | 0.131 |
| <2.6mmol/L | 639 (45.77) | 17/253 | 58/386 | 2.46 (1.43 ~ 4.23) | 0.001 |  |
| ≥2.6mmol/L | 757 (54.23) | 8/300 | 56/457 | 5.10 (2.43 ~ 10.71) | <0.001 |  |
| Anterior wall infarction |  |  |  |  |  | 0.818 |
| No | 757 (54.23) | 11/278 | 61/479 | 3.51 (1.85 ~ 6.68) | <.001 |  |
| Yes | 639 (45.77) | 14/275 | 53/364 | 3.17 (1.76 ~ 5.72) | <0.001 |  |
| AHA lesion types |  |  |  |  |  | 0.091 |
| A/B | 451 (32.31) | 10/157 | 34/294 | 2.04 (1.01 ~ 4.14) | 0.047 |  |
| C | 945 (67.69) | 15/396 | 80/549 | 4.22 (2.43 ~ 7.32) | <0.001 |  |

HR: Hazard Ratio, CI: Confidence Interval

**References**

[1] ARONOW W S. Hypertension guidelines [J]. Hypertension, 2011, 58(3): 347-8.

[2] RUBINO F, NATHAN D M, ECKEL R H, et al. Metabolic Surgery in the Treatment Algorithm for Type 2 Diabetes: A Joint Statement by International Diabetes Organizations [J]. Diabetes Care, 2016, 39(6): 861-77.

[3] SHRANK W H, BARLOW J F, BRENNAN T A. New Therapies in the Treatment of High Cholesterol: An Argument to Return to Goal-Based Lipid Guidelines [J]. Jama, 2015, 314(14): 1443-4.

[4] LIN L, ZHAO J, HU J, et al. Current Smoking has a Detrimental Effect on Survival for Epidermal Growth Factor Receptor (EGFR) and Anaplastic Lymphoma Kinase (ALK) negative Advanced non-squamous Non-small Cell Lung Cancer (NSCLC) Patients Treated with Pemetrexed Continuation Maintenance [J]. J Cancer, 2018, 9(12): 2140-6.

[5] STEVENS P E, LEVIN A. Evaluation and management of chronic kidney disease: synopsis of the kidney disease: improving global outcomes 2012 clinical practice guideline [J]. Ann Intern Med, 2013, 158(11): 825-30.

[6] PRATI F, REGAR E, MINTZ G S, et al. Expert review document on methodology, terminology, and clinical applications of optical coherence tomography: physical principles, methodology of image acquisition, and clinical application for assessment of coronary arteries and atherosclerosis [J]. Eur Heart J, 2010, 31(4): 401-15.

[7] JIN Q H, CHEN Y D, JING J, et al. Incidence, predictors, and clinical impact of tissue prolapse after coronary intervention: an intravascular optical coherence tomography study [J]. Cardiology, 2011, 119(4): 197-203.

[8] LEE S Y, AHN C M, YOON H J, et al. Early Follow-Up Optical Coherence Tomographic Findings of Significant Drug-Eluting Stent Malapposition [J]. Circ Cardiovasc Interv, 2018, 11(12): e007192.

[9] JINNOUCHI H, SAKAKURA K, YANASE T, et al. Impact of stent edge dissection detected by optical coherence tomography after current-generation drug-eluting stent implantation [J]. PLoS One, 2021, 16(11): e0259693.
